# Psilocybin-assisted therapy for severe alcohol use disorder: protocol for a double-blind, randomized, placebo-controlled, 7-month parallel-group phase II superiority trial

**DOI:** 10.1186/s12888-024-05502-y

**Published:** 2024-01-26

**Authors:** Laetitia Vanderijst, Felix Hever, Anne Buot, Charles Dauré, Janaïna Benoit, Catherine Hanak, Johannes Veeser, Margot Morgiève, Salvatore Campanella, Charles Kornreich, Luc Mallet, Christophe Leys, Xavier Noël

**Affiliations:** 1 Laboratory of Medical Psychology and Addictology, Faculty of Medicine, Université libre de Bruxelles, Brussels, Belgium; 2 Research Center for the Promotion of Health, Prosocial Behavior and Wellbeing, Faculty of Psychology, Université libre de Bruxelles, Brussels, Belgium; 3 Sorbonne Université, Institut du Cerveau - Paris Brain Institute - ICM, CNRS, Inserm, Paris, France; 4 Université de Paris, INSERM UMRS1144, 4 avenue de l’Observatoire, 75006 Paris, France; 5 Psychiatric Institute, University Hospital Brugmann, Brussels, Belgium; 6 Université Paris Cité, CNRS, Inserm, Cermes3, F-75006 Paris, France; 7 Département Médical-Universitaire de Psychiatrie et d’Addictologie, Univ Paris-Est Créteil, DMU IMPACT, Hôpitaux Universitaires Henri Mondor - Albert Chenevier, Assistance Publique-Hôpitaux de Paris, Créteil, France; 8 Department of Mental Health and Psychiatry, Global Health Institute, University of Geneva, Geneva, Switzerland

**Keywords:** Psilocybin-assisted therapy, Serotonergic psychedelics, Clinical trial, Severe alcohol use disorder

## Abstract

**Background:**

A significant number of individuals with alcohol use disorder remain unresponsive to currently available treatments, which calls for the development of new alternatives. In parallel, psilocybin-assisted therapy for alcohol use disorder has recently yielded promising preliminary results. Building on extant findings, the proposed study is set to evaluate the feasibility and preliminary clinical efficacy of psilocybin-assisted therapy when incorporated as an auxiliary intervention during inpatient rehabilitation for severe alcohol use disorder. Moreover, it intends to pinpoint the modifications in the two core neurocognitive systems underscored by dual-process models of addiction.

**Methods:**

In this double-blind, randomized, placebo-controlled, 7-month parallel-group phase II superiority trial, 62 participants aged 21–64 years will be enrolled to undergo psilocybin-assisted therapy as part of a 4-week inpatient rehabilitation for severe alcohol use disorder. The experimental group will receive a high dose of psilocybin (30 mg), whereas the control group will receive an active placebo dose of psilocybin (5 mg), both within the context of a brief standardized psychotherapeutic intervention drawing from key elements of acceptance and commitment therapy. The primary clinical outcome is the between-group difference regarding the change in percentage of heavy drinking days from baseline to four weeks posthospital discharge, while safety and feasibility metrics will also be reported as primary outcomes. Key secondary assessments include between-group differences in terms of changes in (1) drinking behavior parameters up to six months posthospital discharge, (2) symptoms of depression, anxiety, trauma, and global functioning, (3) neuroplasticity and key neurocognitive mechanisms associated with addiction, and (4) psychological processes and alcohol-related parameters.

**Discussion:**

The discussion outlines issues that might arise from our design.

**Trial registration:**

EudraCT 2022-002369-14 and NCT06160232.

**Supplementary Information:**

The online version contains supplementary material available at 10.1186/s12888-024-05502-y.

## Background

### Background of the proposed study

Alcohol use disorder (AUD) is a chronic, relapsing mental health disorder defined by compulsive patterns of alcohol seeking that endure despite harmful consequences, inducing clinically significant impairment [1]. AUD has an estimated global prevalence of 5%, with harmful alcohol use causing approximately 3 million deaths worldwide every year, representing 5.3% of global mortality [2]. A substantial proportion of individuals with AUD do not respond to available pharmacological and behavioral treatments: over 50% relapse within the month following alcohol cessation [3], and 70% relapse within the year [4]. This lack of effective treatment modalities underscores the urgent need for innovative therapeutic alternatives.

In parallel, the past decade has witnessed a renewed interest in psychedelic-assisted therapy (PAT). After an initial spike in clinical studies with lysergic acid diethylamide (LSD) in the 1950s and 1960s, prohibition halted all psychedelic research [5]. Following a 25-year hiatus, PAT has re-emerged as a promising treatment option for a range of mental health disorders [6]. Serotonergic psychedelics act as partial agonists of the serotonin 2A receptor (5-HT_2A_R) in the brain [7]. They include LSD, psilocybin (the principal psychoactive component in ‘magic mushrooms’), mescaline (found in certain species of cacti, such as peyote), dimethyltryptamine (DMT, the principal psychoactive compound in the ayahuasca brew), and 5-methoxy-N,N-dimethyltryptamine (5-MeO-DMT, found in various plant and animal species, such as the Sonoran desert toad) [8]. Serotonergic psychedelics induce nonordinary states of consciousness characterized by profound alterations in perception, cognition, and emotion [8]. These compounds present very low long-term toxicity [9, 10], are not associated with addiction [9, 11, 12] and have shown a favorable safety profile when used in clinical settings under therapeutic supervision [13].

Serotonergic psychedelics have demonstrated encouraging results in the treatment of AUD. A contemporary meta-analysis [14] pooled six randomized controlled trials (RCTs; *n* = 536) of PAT for severe AUD (sAUD) conducted in the 1960s. The trial conditions consisted of comparing one single dose of LSD (ranging from 3 mcg/kg: ∼ 210 mcg, to 800 mcg, with a median dose of 500 mcg) to control conditions, including a low dose of LSD (25 or 50 mcg), an alternative placebo (60 mg of d-amphetamine or ephedrine sulfate), time alone to write or treatment as usual, all with equivalent complementary treatment within trials. The meta-analysis demonstrated that participants treated with a single dose of LSD were significantly more likely than those in control conditions to show a reduction in alcohol misuse at the first reported follow-up, ranging from 1 to 12 months posttreatment, with an odds ratio of 1.96 in favor of the LSD treatment. Moreover, treatment comparison indicated a larger reduction in alcohol misuse and greater abstinence rates with LSD treatment than with commonly used AUD medication (i.e., naltrexone, acamprosate, and disulfiram) [14]. More recently, the first contemporary double-blind RCT of psilocybin-assisted therapy for AUD [15], which combined two psilocybin administration sessions with cognitive‒behavioral therapy and motivational interviewing, yielded additional promising results. At the 36-week follow-up, the group that received psilocybin exhibited both a significantly lower percentage of heavy drinking days and a significantly higher rate of abstinence than the group that received a placebo. However, as noted by the authors, the study sample exhibited a lower drinking intensity at screening than that observed in most AUD clinical trials, preventing generalization to populations with a more severe symptomatology. Interestingly, current evidence suggests that PAT might also be effective in treating major depressive disorder [16] and anxiety disorders [17], syndromes that are often comorbid with AUD [18, 19].

These findings pave the way for future research directions to fine-tune our understanding of PAT for AUD. First, experts in the field of PAT research have drawn attention to the need for trials that are more pragmatic in nature [20], i.e., that aim to investigate whether a treatment would have clinically meaningful effects when provided in real-world circumstances, as opposed to trials that are more confirmatory in nature, i.e., that focus on whether a treatment would have any effect in ideal circumstances [21]. Trials leaning toward pragmatism strive to provide the experimental intervention in the realistic conditions under which such treatment will eventually be provided and to reduce bias in participant selection to more closely represent the population that might ultimately seek the experimental treatment. Although both types of trials are necessary to investigate the safety and efficacy of a developing treatment, pragmatic trials, because they are provided closer to ‘real-world’ circumstances, give us more information regarding the feasibility of integrating said treatment within existing care. Trials integrated within real-world clinical pathways also provide access to a participant sample more realistically representing the target population, thereby enhancing the external validity of obtained results [20, 21].

Second, trials targeting the subgroup of individuals exhibiting an AUD defined as severe are necessary, as these patients present a specific profile with impaired psychosocial [22] and neurocognitive functioning [23, 24] and might consequently show a different treatment response than patients with moderate clinical phenotypes. Moreover, individuals with sAUD are particularly prone to repeated relapse [25] and exhibit more psychiatric comorbidities [26] alongside a higher risk of somatic diseases [27], warranting a prioritization in search of better treatment alternatives.

Third, while several authors have proposed diverse mechanisms of action across levels of analysis [28–31], a data-driven comprehension of the mechanisms underpinning PAT’s therapeutic efficacy in the context of AUD remains lacking.

Last, systematic unblinding has been raised as a major threat to the validity of extant findings from ‘double blind’ PAT trials due to high expectancy, presumably resulting in large placebo and nocebo effects in the experimental group and the control group, respectively [32–34]. Studies have used inactive placebos (e.g., [17, 35]), active placebos whose effects considerably differ from the acute effects of serotonergic psychedelics (e.g., [15, 36, 37]), or doses of psilocybin presumably too low to maintain blinding (e.g., [38, 39]), leading to a general call for more effective active placebo conditions and a systematic reporting of blinding efficacy [32–34].

### Psilocybin-assisted therapy for severe alcohol use disorder: potential mechanisms of action

Below, we present a nonexhaustive selection of proposed therapeutic mechanisms that our study aims to interrogate. In considering multilevel approaches, the integrative view, which aims toward integrating all levels in one comprehensive theory, contrasts with the pluralistic view, which postulates that diverse complementary pathways must be considered to achieve an exhaustive understanding of a given phenomenon [40]. Although the elegant simplicity of an overarching theory is appealing, the multifactorial nature of psychiatric disorders [41], combined with our currently limited understanding of PAT’s mode of action and the possibility that therapeutic mechanisms differ across individuals receiving treatment [40], encourages us to adopt a pluralistic approach in our proposed research.

#### Cortical neuroplasticity

Substance use disorders have been defined as ‘diseases of learning and memory’ [42], whereby drugs of abuse usurp the neural mechanisms underlying these processes by impairing neuroplasticity. This long-term dysregulation has been hypothesized to underpin the learning difficulties observed in patients with sAUD, thereby constituting a major challenge to fostering beneficial change through psychotherapy [29].

Preclinical studies suggest that serotonergic psychedelics belong to the class of psychoplastogens, i.e., compounds capable of rapidly promoting neuroplasticity in cortical neurons (see [43] for a review). It has been suggested that these properties may represent a key mechanism underlying the efficacy of serotonergic psychedelics in the treatment of psychiatric disorders [44, 45], whereby psychedelic-induced neuroplasticity may open a window of opportunity for therapeutic learning and associated beneficial behavioral change [43]. However, there is little direct evidence of psilocybin-induced cortical plasticity in humans, with no evidence in individuals with AUD, and the relationship between psychedelic-induced cortical neuroplasticity and clinical outcomes remains to be established [43].

#### Neurocognitive mechanisms: a dual-process approach

According to the Impaired Response Inhibition and Salience Attribution (I-RISA) dual-process model of addiction [46], problematic alcohol use results from a disturbed balance between 2 interacting neurocognitive systems: a strengthened striatum-dependent reward system and a weakened prefrontal cortex-dependent reflective system. As addiction develops and exposure to alcohol intensifies, successive neuroadaptations in these interconnected cerebral networks lead to overactivation of the reward system in response to alcohol-related stimuli, generating craving and an automatic approach tendency toward alcohol cues, while alcohol-induced neurotoxicity precipitates alterations in prefrontal structures involved in cognitive control, disabling the voluntary inhibition of prepotent responses and a flexible adaptation to a changing environment [47].

Preliminary preclinical evidence suggests that psychedelics’ psychoplastogenic properties [44] might directly counteract the hypofrontality observed in chronic alcohol exposure [48], thereby restoring the function of the reflective prefrontal system and its regulatory action on the reward system. Several studies established a causal role for reduced prefrontal metabotropic glutamate receptor 2 (mGluR2) function in craving and relapse responses in preclinical models of alcohol dependence [48–52]. More recently, a single administration of psilocybin was found to restore mGluR2 expression and reduce operant alcohol-seeking behaviors in alcohol-dependent rats [48]. However, to our knowledge, there are no published data to date regarding the neurobiological mechanisms associated with PAT in individuals with sAUD. Consequently, the direct influence of PAT on key functions outlined by the I-RISA dual-process model of addiction, namely, alcohol cue reactivity and inhibitory control, and their role in clinical improvement remain unknown.

#### Psychological mechanisms - role of the acute psychedelic experience

Across psychiatric diagnoses, PAT clinical trials have suggested a relationship between the quality of the acute psychedelic experience and clinical ameliorations [36, 53, 54]. Several dimensions predicted improvement, of which the majority are associated with the ‘mystical’ quality of the experience, which is characterized by feelings of unity, sacredness, ineffability, a noetic quality, transcendence of time and space, and a deeply felt positive mood [55]. Researchers have also proposed alternative putative mediators of efficacy related to the acute psychedelic effects, including operant conditioning of acceptance (‘learning to let go’ [56]) or the opportunity to access and process deep traumatic memories otherwise unbearable and/or inaccessible (the “helioscope” hypothesis [57]).

#### Psychological mechanisms - psychological flexibility

The concept of psychological flexibility, which was initially developed within the acceptance and commitment therapy (ACT) framework, is defined as ‘the ability to contact the present moment more fully as a conscious human being, and to change or persist in behavior when doing so serves valued ends’ [38, p.7]. Augmented psychological flexibility is hypothesized to facilitate reductions in alcohol abuse by enhancing one’s capacity to navigate distressing experiences without resorting to escape or avoidance while concurrently bolstering motivation to change [58]. Two retrospective survey studies suggest that increased psychological flexibility might mediate the therapeutic effects of the quality of the psychedelic experience on depression and anxiety on the one hand [59] and of psychedelic therapy on alcohol consumption and posttraumatic stress symptoms on the other hand [60]. Of note, the psychotherapeutic intervention accompanying the psilocybin session in our study will draw from key elements of ACT. The intervention and its rationale are described in the methods section.

#### Psychological mechanisms - connectedness

Increased connectedness to oneself, others, and the world has been proposed as a key psychological mechanism underlying PAT’s transdiagnostic efficacy [61–64]. In qualitative interviews, participants identified this tri-dimensional factor as a key element underlying the therapeutic effect of PAT [61, 62], including reduced craving and substance use in individuals with addiction [62].

#### Psychological mechanisms - alcohol-related parameters

The first contemporary pilot trial of PAT for AUD [54] revealed decreased craving for alcohol and both increased motivation to change alcohol consumption behavior and abstinence self-efficacy following PAT. While encouraging, these effects need to be replicated in RCTs with larger samples.

### Proposed study

Here, we present the protocol for an RCT aiming to determine the feasibility and preliminary clinical efficacy of PAT as a complementary intervention during inpatient rehabilitation for sAUD and to characterize associated changes in the two key neurocognitive systems identified by dual-process models of addiction. We strive to address the aforementioned open questions and limitations by, first, conducting a ‘pragmatic’ trial that integrates PAT within an existing model of care, i.e., the classic four-week inpatient rehabilitation program proposed in a public university hospital, to assess the feasibility and efficacy of this intervention when provided in close-to real-world circumstances; second, defining our population to only include patients with an AUD defined as severe according to the Diagnostic and Statistical Manual of Mental Disorders, Fifth Edition, Text Revision (DSM-5-TR; 6 criteria or more [65]); and, third, proposing an active placebo condition aimed at maintaining blinding, while systematically assessing and reporting blinding efficacy in participants and therapists.

Our primary hypotheses are as follows:

- A greater decrease in the percentage of heavy drinking days will be observed among participants receiving a high dose of psilocybin (30 mg, high dose group) than in those receiving an active placebo (5 mg psilocybin, active placebo group) from baseline to four weeks posthospital discharge.
- Both treatment arms will not differ in terms of serious adverse reactions during the course of the trial.

Furthermore, our secondary clinical and mechanistic hypotheses are as follows:

- The high-dose group will show a greater improvement in a range of alcohol consumption parameters than the active placebo group (↓ % of heavy drinking days at 6 months posthospital discharge, ↓ drinks per day and ↑ % of days of abstinence at 4 weeks and 6 months posthospital discharge).
- The high-dose group will demonstrate greater reductions in depression, anxiety, and subsyndromal trauma symptoms, as well as a greater improvement in health-related quality of life compared to the active placebo group.
- The high-dose group will demonstrate greater increases in psychological flexibility and connectedness than the active placebo group. These increases will correlate with clinical outcomes.
- The high-dose group will show a greater increase in neuroplasticity compared to the active placebo group, as measured with the EEG-derived auditory long-term potentiation (LTP) paradigm [66]. This increase will correlate with clinical outcomes.
- The high-dose group will show a greater improvement in alcohol-related parameters than the active placebo group (↑ abstinence self-efficacy, ↑ motivation to change, ↓ craving). These improvements will correlate with clinical outcomes.

Exploratory outcomes:

- Modulations of EEG-derived measures of alcohol cue reactivity and inhibition will be compared across groups, and their relationship with clinical outcomes will be investigated.
- We will explore the impact of the quality of the acute psilocybin journey, in terms of experiential avoidance vs. acceptance, mystical experience, and trauma processing, on clinical outcomes.
- We will explore the impact of the therapeutic alliance on the quality of the psilocybin experience and on clinical outcomes.
- We will explore the impact of psilocybin on self-reported interoception and its associations with clinical outcomes.

Blinding and expectancy will be assessed systematically.

## Methods

### Trial design and study setting

The proposed trial is a monocentric, randomized, double-blind, placebo-controlled, 1:1 parallel-group 7-month clinical trial comparing the effects of a high dose of psilocybin (30 mg) to those of a low dose of psilocybin (5 mg, active placebo) in 62 participants with sAUD. The trial will be conducted at the Psychiatry Department of the Brugmann University Hospital. Recruitment will start in winter 2024, and we expect completion of the study in autumn 2026. Our protocol adheres to the SPIRIT guidelines (Standard Protocol Items: Recommendations for Interventional Trials [67]).

### Trial population

Sixty-two participants will be included, with 31 participants receiving the experimental treatment (psilocybin high dose: 30 mg) and 31 participants receiving the active placebo (psilocybin low dose: 5 mg).

#### Sample size

In line with extant studies indicating medium to large effects of PAT on alcohol consumption in patients with AUD (Cohen’s d = 1, [54]; Hedges’ g = 0.52 [15]), the sample size was calculated to observe a medium-size effect (Cohen’s f = 0.25) with a power of 0.9 and a type I error set at 0.05. A priori power calculation based on the primary clinical hypothesis (mixed-design analysis of variance, two groups: high dose vs. active placebo; two timepoints: % heavy drinking days from 8 weeks prehospitalization to 1 day prehospitalization vs. % heavy drinking days from hospital discharge to 4 weeks posthospital discharge) indicated a needed sample size of *n* = 46. Accounting for 25% attrition, we set the total sample size to *n* = 62. If the drop-out rate is higher, we will continue to include participants until 46 have completed visit 14 (primary clinical outcome).

#### Screening and eligibility

Candidate participants must provide written informed consent before being screened for eligibility. The screening process will consist of a neuropsychiatric screening and a physical screening taking place across two visits. Alcohol consumption will be recorded at both visits using the Timeline Follow-Back method (TLFB; [68]) to obtain a baseline measure. The final decision on eligibility will be made by medical doctors only.

Candidate participants will have to comply with the following key eligibility criteria:

Key inclusion criteria

- Age of 21–64 years,
- BMI between 17.5 and 30 kg/m^2^,
- Desire to stop or decrease drinking,
- Have a diagnosis of severe alcohol use disorder (sAUD), according to the DSM-5-TR (6 criteria or more),
- Undergoing a 4-week alcohol detoxification program at the Brugmann University Hospital,
- Women of childbearing potential must be using an effective, established method of contraception from inclusion until four weeks posthospital discharge (5 weeks post-psilocybin administration),
- Men with a woman of childbearing potential should use a condom during intercourse from inclusion until four weeks posthospital discharge (5 weeks post-psilocybin administration).

Key exclusion criteria

- Cardiovascular, hepatic, gastroenterological, hematologic, renal, endocrine, metabolic, inflammatory, or neurological diseases or any other somatic condition that, in the opinion of the medical investigator, would pose a risk to the participant’s participation in the study,
- Serious abnormalities of complete blood count or chemistries, biological abnormalities including TP < 50%, albumin < 35 g/L, total bilirubin > 35 µmol/L, leading to a Child B or C score,
- Cognitive impairment (Folstein Mini Mental State Exam [69] score < 26),
- Alcohol withdrawal complication(s), seizure, head injury or stroke within the last 6 months,
- Current active acute stress disorder/posttraumatic stress disorder,
- Lifetime history of schizophrenia spectrum disorders, other psychotic disorders, or bipolar spectrum disorders,
- Significant risk of suicide according to clinician assessment,
- Family history of schizophrenia spectrum disorders, other psychotic disorders, or bipolar I disorder in first- or second-degree relatives,
- Other substance use disorder (except for caffeine, nicotine, or cannabis) according to DSM-5-TR criteria in the two months preceding inclusion,
- Severe cannabis use disorder according to the DSM-5-TR,
- Need to take medication with significant potential to interact with psilocybin,
- Pregnancy and breastfeeding, at screening visit and until dosing day.

### Clinical intervention

Our trial will compare a single administration of either 30 mg of psilocybin (high dose) or 5 mg of psilocybin (active placebo) embedded in a supportive psychotherapy protocol integrating key elements of ACT [70] within the context of a four-week inpatient alcohol detoxification program. Pharmaceutical-grade psilocybin is provided in the form of capsules by Psilo Scientific Ltd. (Wholly Owned Subsidiary Of Filament Health Corp.).

#### Dose rationale

The optimal therapeutic dose of psilocybin in the treatment of sAUD has not been determined. While in the only published contemporary RCT of PAT for AUD, a second dose of up to 40 mg/70 kg was administered (following a first dose of 25 mg/70 kg, [15]), all currently ongoing trials in this population have opted for one to two doses of 25 mg (NCT04141501, NCT05416229, NCT05646303, NCT04620759, and NCT04410913). Of note, relative doses have been mostly substituted with absolute doses, following evidence that body weight has no influence on psilocybin pharmacokinetics [71] and subjective effects [72] in humans. There may be some advantages to selecting a higher dose of 30 mg. First, preclinical evidence suggests a dose-dependent effect on increases in the expression of some neuroplasticity-related genes induced by serotonergic psychedelics [73–75], a key proposed therapeutic mechanism [44, 45]. Second, phase I and II studies suggest a dose-dependent effect on the quality of the psychedelic experience [54, 76], with high mystical experience scores associated with 30 mg/70 kg of psilocybin [76], another proposed therapeutic mechanism [77]. Last, early studies with LSD and contemporary studies with psilocybin suggest that patients with AUD may tend to display more tolerance to serotonergic psychedelics, whereas high doses of 30–40 mg/70 kg are well tolerated in this population [15, 54, 78].

#### Explanation for choice of comparator

We decided to opt for a design in which the control group receives a low yet psychoactive dose of psilocybin (5 mg, [76]), which would theoretically not induce significant clinical benefits (e.g., [38] observed a significant decrease in depression symptomatology in participants with treatment-resistant depression after a single 25 mg dose relative to a 1 mg dose, but not with a 10 mg dose), while it may adequately maintain blinding (5 mg of psilocybin was shown to induce subjective changes significantly greater than those of an inert placebo; [76]). A complementary measure to enhance blinding will consist of an emphasis on the substantial interindividual and intersituational variability in response to psilocybin to increase ambivalence. Last, blinding efficacy (participants, therapists) and expectancy (participants) will be assessed and reported, as recommended in the literature [32–34].

#### Benzodiazepine discontinuation

Benzodiazepines with a short half-life (lorazepam [79]) will be used for the treatment of acute alcohol withdrawal syndrome upon admission in the inpatient alcohol detoxification program. The shorter half-life allows us to minimize the potential benzodiazepine-psilocybin interaction by ensuring a relatively lower lorazepam plasma concentration rapidly after full tapering off (at least 3 days before psilocybin administration), which is relevant, as benzodiazepines might blunt the psychedelic experience. The Clinical Institute Withdrawal Assessment Alcohol Scale Revised (CIWA-AR; [80]) will be used to assess withdrawal symptoms to ensure safe progressive lorazepam discontinuation, in line with evidence-based practice guidelines recommending individualized tapering off schedules with withdrawal scales [81]. In case full tapering off from lorazepam cannot be achieved within 17 days due to withdrawal symptoms, participants will be excluded from the study.

#### Behavioral intervention

Both treatment arms will be provided in the context of a three-part psychotherapeutic intervention, involving two preparation sessions, support during the psilocybin dosing session, and two integration sessions, all conducted during the four-week inpatient care. The sessions will be conducted by a mixed-gender therapist dyad. The leading therapist must hold a master’s degree in a related field (psychiatry, psychology) and a minimum of three years of experience with psychiatric patients in a clinical setting. The co-therapist must hold a master’s degree in a related field or be in their ultimate or penultimate year in pursuing such a degree. All therapists will be required to read the entire study manual, attend a study-specific training workshop, and attend supervision sessions by a designated lead therapist.

Although contemporary clinical trials have invariably provided preparation and integration sessions before and after psilocybin administration, respectively, the theoretical orientation and frequency of these sessions have varied across trials (see [82] for a review). Relatedly, the optimal model to maximize PAT efficacy is yet to be clarified. For our study, we decided to opt for a theory-driven, manualized procedure that incorporates principles of ACT, an approach with demonstrated efficacy in the treatment of AUD [83, 84]. Our manual is adapted from the Yale Manual for Psilocybin-Assisted Therapy of Depression [85] to fit our target population (patients with sAUD) and our study design.

ACT integrates radical behaviorism with experiential methods to address transdiagnostic factors that contribute to psychological suffering, including fusion with thoughts, evaluation of experiences, avoidance, and reason-giving [86]. To transcend these psychological processes, ACT aims to foster psychological flexibility through six key processes: present moment awareness, acceptance, defusion, value clarification, committed action, and the development of a flexible sense of self [86]. The rationale for integrating ACT principles into the PAT model stems from the proposed concordance between mechanisms of change generated by both approaches, as the psychedelic experience may provide direct experiential access to key ACT processes known to enhance psychological flexibility (see [87] for an in-depth discussion).

#### Contextual considerations

The psychedelic experience comes with an increased sensitivity to internal and external stimuli. It follows that the general context of psychedelic administration fundamentally shapes drug response and hence safety, as well as potentially therapeutic efficacy [9, 88]. Contextual factors are commonly labeled as *the set and setting* [88]. The set refers to the participant’s mindset and includes the readiness, expectations, and intentions that one brings to the psychedelic experience, as well as any other preexisting psychological factors, including personality and psychopathology. The setting comprises the physical, social, and cultural environment surrounding the psychedelic experience. As soon as screening occurs, the first aspect of the set to be systematically considered in PAT trials consists of the psychiatric background of candidate participants. As mentioned in the study’s key exclusion criteria, individuals with a lifetime history of psychotic or bipolar disorders or with a family history of these conditions in first- or second-degree relatives are currently excluded from participation to prevent the risk of inducing a psychotic or a manic episode in those with these conditions or with a potential genetic vulnerability [9]. Further aspects of the set are addressed in the preparation sessions, whereas the setting is given specific attention during psychedelic administration. More details are provided below.

#### Preparation sessions

The main function of the sessions leading to psilocybin administration is to prepare participants for exposure to the substance [89] in terms of expectations, assumptions, readiness and intentionality [9, 88]. Our study includes two preparation sessions prior to the psilocybin administration session. The first session will take place two weeks into the inpatient alcohol detoxification program, and the second session will take place one day before the drug administration session (visit 6, day 20 +/-2). We provide the first preparation session two weeks into the detoxification program so that participants have already gone through the most intense withdrawal symptoms and have almost entirely tapered off from the benzodiazepines prescribed to manage these symptoms.

The principal aims of the two preparation sessions are as follows [85]:

- To develop a therapeutic alliance between participants and therapists,
- To listen to the participant’s narrative of sAUD and treatment history to understand patterns of psychological inflexibility that are most prominent,
- To provide psychoeducation regarding psilocybin and its acute effects,
- To delineate the therapeutic boundaries, including regarding the use of therapeutic touch,
- To outline safety measures, including the rescue medications that can be used if necessary,
- To teach and practice grounding techniques, including diaphragmatic breathing, therapeutic touch and body scanning,
- To provide psychoeducation regarding the cognitive processes and behaviors that contribute to inflexibility from an ACT perspective and how one can shift from these patterns of inflexibility and associated maladaptive coping strategies toward processes that foster psychological flexibility through an interaction between the principles of ACT and the psilocybin experience,
- To assist the participant in setting an intention for the psilocybin session with the help of value cards to first identify important values.

#### Psilocybin dosing session

Psilocybin will be administered in a specifically dedicated room in the Brugmann University Hospital, decorated in a way that creates a warm and home-like atmosphere rather than a sterile clinical setting, thereby maximizing participant comfort [9]. The psilocybin session will be video-recorded for training (supervision) and safety purposes.

Participants will be dosed per os and receive either a high dose of psilocybin (30 mg) or a low dose of psilocybin (5 mg - active placebo) as determined by computer-generated randomization. They will be invited to lie comfortably on a bed, wearing eyeshades and headphones, and instructed to direct their attention toward their internal experience and ‘trust, let go, be open’ [90]. Through headphones, participants will be listening to a standardized music playlist, specifically curated to accompany the three principal intensity phases of the experience: the onset of psychoactive effects, the peak plateau, and the return to an ordinary state of consciousness [91].

After psilocybin administration, participants will remain under observation for 6–8 h, based on the drug’s duration of action [92]. Vital signs will be measured at 30 min intervals for the first 2 h and every hour thereafter until session completion. The assigned therapist-dyad will remain present throughout, and interactions with the participants will be supportive and nondirective.

Psychedelics generally intensify emotional experience and may give rise to anxiety, dysphoria, fear, panic, or paranoia [9]. Across PAT trials, the first response to psychological distress has been psychological support and reassurance, as well as therapeutic touch such as hand holding, according to preestablished individual boundaries. Participants may also be redirected to other grounding techniques practiced during the preparation sessions, namely, body scan and diaphragmatic breathing. Psychological support has generally been sufficient to address transient psychological distress across contemporary trials [13, 15, 35]. However, if insufficient, rescue medication will be available for one-time administration to treat anxiety [Temesta Expidet^TM^ (Lorazepam) 1-2.5 mg, sublingual] and acute psychosis posing a danger to the participant or others [Zyprexa Velotab^TM^ (Olanzapine) 5–10 mg sublingual]. Hypertensive urgency will be treated with captopril, 25–100 mg per os. Emergency treatment will be available on site.

#### Integration sessions

The psychotherapeutic sessions that follow the psilocybin experience aim to support its integration, “the process by which a psychedelic experience translates into positive changes in daily life” ( [93], p. 8). The therapists assist the participants in making sense of the psilocybin experience and deriving insights and learnings that can be implemented in their lives to improve mental health and general wellbeing, including reductions in drinking behavior. Our study includes two integration sessions, one day and one week after the psilocybin session.

The principal aims of the two integration sessions are as follows [85]:

- To elicit a narrative of the participant’s psilocybin experience,
- To discern and examine facets of the participant’s experience that align with ACT principles, as well as occasions when the participant shifted closer to or farther from psychological flexibility,
- To further clarify values that are central for the participant by integrating the emerged material,
- To reflect on the extent to which the participant currently lives according to these values and on actionable ways to further reinforce committed action,
- To explore actionable ways to develop functional reinforcing coping mechanisms that may replace drinking behavior.

#### Concomitant care

The care as usual trajectory offered within the context of the inpatient alcohol detoxification program involves two weekly group therapy sessions around relapse prevention and psychosocial support and a one-time group therapy session around the influence of alcohol consumption on close relationships.

Concomitant interventions will be assessed at trial entry and during the study to control for confounds on study endpoint measures. Except for the use of lorazepam for the treatment of acute alcohol withdrawal syndrome, concomitant pharmacotherapy for sAUD is not allowed.

### Outcomes

#### Primary clinical outcome

The primary clinical outcome is the difference between the two treatment arms in terms of change from baseline (8 weeks to 1 day pre-enrollment) to 4 weeks posthospital discharge (1 day to 4 weeks postdischarge) in the percentage of heavy drinking days, defined as ≥ 5 standard drinks/60 g of alcohol for males and ≥ 4 standard drinks/48 g of alcohol for females [15]. A reduction in heavy drinking days yields clinically significant health ameliorations [94], matches the treatment objectives of numerous patients [95] and is recognized by the European Medicines Agency as an adequate measure of efficacy [96]. Moreover, previous results suggest that PAT might lead to a decrease in alcohol consumption without necessarily inducing complete abstinence [15, 54]. We opted for a primary clinical outcome measure at four weeks posthospital discharge to limit attrition, as well as because the literature suggests that at least 50% of patients resume excessive heavy drinking within the first month following an inpatient detoxification program [3, 97]. Longer term follow-up data will be collected up to six months posthospital discharge (see secondary outcomes). The TLFB [68] will be used to measure alcohol consumption. The TLFB is a semi-structured interview designed to assess daily alcohol (and other drug) use by obtaining estimates of quantity, frequency, and usage patterns within a specific timeframe. The tool has demonstrated adequate to excellent reliability and validity [98, 99]. See Table 1 for an overview of key outcomes and sampling time points.

#### Primary feasibility and safety outcomes

For full transparency on feasibility, we will report on both inclusion and retention rates. A consort diagram illustrating the participant retention rate during the recruitment, screening, and data collection stages will be presented. Demographic information and reasons for exclusion will be reported.

 [100] have drawn attention to a potential underreporting of adverse events in PAT studies due to a lack of systematic assessment and disclosure. To address this limitation, safety has been added as a primary outcome in our trial. Adverse events will be systematically recorded at each study visit and follow-up and reported in trial publications. They will be classified according to severity, causality, and whether they were expected or not.

#### Secondary clinical outcomes

The difference between both treatment arms with respect to change from baseline to 4 weeks (*) and six months (^▲^) posthospital discharge in terms of:

- The percentage of heavy drinking days (^▲^) measured with the TLFB;
- The number of drinks per day (*,^▲^) measured with the TLFB;
- The percentage of days of abstinence (*, ^▲^) measured with the TLFB;
- Phosphatidyl-ethanol (PEth) blood concentration (*), which will serve as an objective marker of alcohol consumption [101] to corroborate the self-reported drinking data.
- As symptoms of anxiety, mood disorders, and trauma frequently co-occur with alcohol use disorders [18, 19, 102], we will assess changes in depression, anxiety, and trauma symptoms (*,^▲^) using the Beck Depression Inventory (BDI; [103]), the State-Trait Anxiety Inventory (STAI; [104], trait subscale), and the International Trauma Questionnaire (ITQ; [105]), respectively.
- To obtain a more global view of recovery, we will utilize a Patient Reported Outcome Measure (PROM), the Substance Use Recovery Evaluator (SURE; [106]) (*,^▲^), which assesses recovery from (drug and) alcohol dependence through a holistic lens by including the dimensions of drinking and drug use, self-care, relationships, material resources and outlook on life.

#### Neurocognitive mechanisms

The difference between both treatment arms with respect to change from baseline to one day and one week postpsilocybin dosing in terms of:

- Neuroplasticity as assessed with the EEG-derived auditory long-term potentiation paradigm [66].
- EEG-derived alcohol cue reactivity [107].
- EEG-derived alcohol cue inhibition [107].

#### Acute psychedelic effects

To quantitatively characterize the acute psychedelic effects and their possible relationship to clinical outcomes, we will use the following scales:

- The Revised Mystical Experience Questionnaire-30 items (MEQ30; [108]) to assess the mystical quality of the psychedelic experience according to 4 dimensions: unity/sacredness/noetic quality (also referred to as the ‘mystical dimension’), positive mood, transcendence of time & space, and ineffability.
- The Acceptance/Avoidance-Promoting Experiences Questionnaire (APEQ; [109]) to assess the acute effect of psychedelic drugs on the ACT construct of psychological flexibility by measuring acceptance- and avoidance-related experiences.
- The Helioscope Questionnaire (Hasler, in validation) to investigate trauma access and processing under psychedelics.

#### Psychological processes and alcohol-related parameters

The difference between both treatment arms with respect to change from baseline to one week post-psilocybin administration, as well as four weeks, three and six months posthospital discharge on the following scales:

- The Acceptance and Action Questionnaire II (AAQII; [110]) to assess the construct of psychological (in)flexibility.
- The Multidimensional Assessment of Interoceptive Awareness (MAIA; [111]) to evaluate interoceptive processes across 8 dimensions: noticing, trusting, not distracting, not worrying, attention regulation, emotional awareness, self-regulation, and body listening.
- The Watts Connectedness Scale (WCS; [63]) to measure felt connectedness to self, others, and the world.
- The Penn Alcohol Craving Scale (PACS; [112]) to assess the frequency, intensity, and duration of past-week cravings.
- The Alcohol Abstinence Self-Efficacy Scale (AASE; [113]) to evaluate participants’ confidence in their ability to resist drinking alcohol in high-risk situations.
- Readiness to change alcohol consumption behavior will be measured by collecting Likert-scale ratings of the participants’ (1) perception of the importance of change in drinking; (2) confidence in their ability to change; (3) readiness for change and (4) commitment to the goal of abstinence [15].

#### Expectancy, blinding, and therapeutic alliance

Participants’ expectancy will be assessed with the Stanford Expectations of Treatment Scale (SETS; [114]) at baseline, and blinding efficacy will be assessed by asking participants and therapists to guess group allocation and indicate guess certainty following the psilocybin administration session. Furthermore, we will assess therapeutic alliance in both participants and therapists after the last preparation session with the Working Alliance Inventory-Short Revised (WAI-SR; [115]).

**Table 1:** Schedule of key study activities

	Screening	Baseline	Preparation 1	Preparation 2	Dosing	Integration 1	Integration 2	4-week endpoint	6-month follow-up
**Days relative to dosing**		-20	-7	-1	0	+ 1	+ 7	+ 35	+ 190
**Key assessments**									
Informed consent	X								
Psychiatric screening	X								
Physical screening	X								
**Key outcomes**									
*Clinical outcomes*									
Heavy drinking		X						X	X
Drinking days		X						X	X
Abstinence		X						X	X
PEth								X	
Mood		X		X			X	X	X
Anxiety		X		X			X	X	X
Trauma		X		X			X	X	X
Global functioning		X						X	X
*Safety*									
Adverse events		X	X	X	X	X	X	X	X
*Feasibility*									
Insclusion rates	X								
Retention rates			X	X	X	X	X	X	X
*Neurocog. outcomes*									
Neuroplasticity (LTP)			X			X	X		
Cue reactivity			X			X	X		
Inhibition			X			X	X		
*Drug experience*									
Mystical (MEQ)					X				
Acceptance/Avoidance (APEQ)					X				
Trauma access/process. (Helioscope Q.)					X				
*Psychological processes*									
Flexibility (AAQQ II)		X		X			X	X	X
Interoception (MAIA)		X		X			X	X	X
Connectedness (WCS)		X		X			X	X	X
*Alcohol-related parameters*									
Craving (PACS)		X		X			X	X	X
Abstinence self-efficacy (AASE)		X		X			X	X	X
Readiness to change		X		X			X	X	X

*Abbreviations: PEth* Phosphatidylethanol, *LTP* Long-term potentiation, *MEQ* Mystical Experience Scale, *APEQ* Acceptance/Avoidance-Promoting Experiences Questionnaire, *AAQ-II* Acceptance and Action Questionnaire Version 2, *MAIA* Multidimensional Assessment of Interoceptive Awareness, *WCS* Watts Connectedness Scale, *PACS* Penn Alcohol Craving Scale, *AASE* Alcohol Abstinence Self-Efficacy Scale

### Recruitment

The primary source of participants will consist of individuals who attend a preadmission consultation to enroll in the inpatient alcohol detoxification program at the Brugmann University Hospital. The study protocol will be presented to these patients, who may then attend a screening visit (eligibility screening 1) with an investigator and a therapist after signing the informed consent form.

Additional recruitment venues will consist of referrals from local inpatient and outpatient psychiatric units and local and national referrals from physicians.

### Data analysis

The data will be analyzed according to a statistical analysis plan that will be uploaded on ClinicalTrials.gov before unmasking. Significance levels for all analyses will be set at 0.05. Clinical outcome analyses (alcohol consumption parameters) will be performed on the intention-to-treat population, including all randomized patients who have completed the dosing session (visit 7). Changes in continuous outcomes will be analyzed with mixed-model analysis of variance with Helmert contrasts when indicated. For time series analysis, general linear mixed models will be used in the case of missing data. Mediation analyses will be used to evaluate whether hypothesized mechanistic variables mediate the effect of treatment on clinical improvements, for example, whether the mystical experience score during psilocybin administration mediates the impact of treatment on changes in the percentage of heavy drinking days. Treatment-emergent adverse events will be classified, tabulated, and treatment groups contrasted using Chi squared or Fisher’s exact test.

## Discussion

Although our study aims to address limitations that have been raised within PAT research while investigating questions that currently remain unanswered, some issues might arise from our design. First, the choice to opt for an active placebo group that receives a low yet psychoactive dose of 5 mg of psilocybin involves some risks. Although we hope to maintain blinding or at least increase group allocation ambivalence and therefore reduce the bias induced by large placebo and nocebo effects in the experimental and control groups, respectively, we might be unsuccessful. It is unclear to what extent the effects of a low dose might be mistaken for those of a high dose, and this might depend on individual factors such as past psychedelic experience and sensitivity to psychedelic effects. Moreover, our approach might decrease the effect sizes pertaining to group differences, as even low doses of psilocybin show considerable occupancy of cortical 5-HT_2A_Rs [116] and may therefore induce a therapeutic effect.

Second, while our trial aims to be pragmatic and include a more representative, diverse pool of participants by recruiting directly from within the hospital, we must adhere to safety guidelines and respect strict and rather restrictive inclusion and exclusion criteria, which limits the range of individuals we can include.

Third, as we propose the experimental treatment integrated within a real-world clinical pathway, participation may influence patients’ relationships with other inpatients not included in the study, which might impact social relations during detoxification, thereby affecting treatment trajectory.

Last, our study includes a single dosing session, which might not be enough. Contemporary clinical studies have involved one to three dosing sessions (e.g., [38, 39, 117]), with two psilocybin sessions proposed in the only RCT of PAT for AUD [15]. Interestingly, important decreases in the number of heavy drinking days were already observed after the first session, suggesting a therapeutically significant effect of a single administration. An ongoing trial compares the clinical efficacy of one versus two dosing sessions on major depressive disorder with co-occurring AUD (NCT04620759), which will inform the development of future interventions.

### Electronic supplementary material

Below is the link to the electronic supplementary material.

Supplementary Material 1

## Data Availability

No datasets were generated or analysed during the current study.

## Abbreviations

ACT: Acceptance and Commitment Therapy
AUD: Alcohol Use Disorder
CIWA-AR: Clinical Institute Withdrawal Assessment Alcohol Scale Revised
DMT: Dimethyltryptamine
DSM-5-TR: Diagnostic and Statistical Manual of Mental Disorders, Fifth Edition, Text Revision
I-RISA: Impaired Response Inhibition and Salience Attribution
LSD: Lysergic Acid Diethylamide
LTP: Long-Term Potentiation
mGluR2: Metabotropic Glutamate Receptor 2
PAT: Psychedelic-Assisted Therapy
PEth: Phosphatidyl-ethanol
PROM: Patient Reported Outcome Measures
RCT: Randomized Controlled Trial
sAUD: Severe Alcohol Use Disorder
SPIRIT: Standard Protocol Items:Recommendations for Interventional Trials
TLFB: Timeline Follow-Back
5-HT2AR: Serotonin 2A Receptor
5-MeO-DMT: 5-Methoxy-N,N-dimethyltryptamine
